# A comprehensive review of sleep disordered breathing in people with congenital heart disease

**DOI:** 10.3389/frsle.2026.1857984

**Published:** 2026-08-17

**Authors:** Kate Maxfield, Noah Weaver, Michelle Wei, Daniel Matloff, Meghana Partha, Michael D. Seckeler, Daniel Combs

**Affiliations:** 1 Center for Sleep, Circadian and Neuroscience, University of Arizona, Tucson, AZ, United States; 2 Department of Pediatrics, University of Arizona, Tucson, AZ, United States

**Keywords:** cognition, congenital heart disease, obstructive sleep apnea, quality of life, sleep disordered breathing, sleep disorders

## Abstract

**Introduction:**

Sleep disordered breathing (SDB) is known to be a comorbidity associated with congenital heart disease (CHD). This review evaluates the role of sleep disorders as a contributing factor to impairments in cardiovascular health, neurocognitive function, and health-related quality of life in individuals with CHD.

**Methods:**

A narrative review was conducted across PubMed, Ovid, and Cochrane Library (1990–2025) searches for studies cross-referencing “congenital heart disease” or “Fontan circulation” with sleep-related terms. Inclusion criteria were studies in CHD populations using objective or self-report sleep assessments; exclusions included non-English, non-original data, or conference abstracts. References of included articles were also screened to identify additional potentially relevant articles. Thirty three of 523 articles screened met inclusion criteria.

**Results:**

SDB is prevalent in both children and adults with CHD, with reported rates ranging from 31 to 63% in adults and up to 57% in children. Both obstructive and central sleep apnea are observed. Comorbid SDB is associated with increased inpatient mortality in infants, poorer quality of life in children and adults, neurocognitive impairments, and increased cardiovascular risk including arrhythmias and paradoxical emboli. Evidence for effective treatment remains limited, though lifestyle, behavioral, and ventilation strategies show potential benefit.

**Discussion:**

SDB is common in both children and adults with CHD and has a negative association with behavior, cognition and quality of life for people with CHD.

## Introduction

Due to improved surgical techniques and medical practices, the life expectancy of individuals with congenital heart disease (CHD) has increased significantly over the past several decades. Currently, around 97% of infants with CHD survive to age 18, and, 75% live to age 60 years (Dellborg, 2024). Two thirds of the population with CHD are adults now (Dellborg, 2024). Despite these improvements in life expectancy, people with CHD experience various long-term comorbidities. CHD is associated with increased risk and younger age of onset for cardiovascular diseases, especially heart failure and stroke (El-Chouli et al., 2023). Arrhythmias are also prevalent among individuals with CHD, which predisposes them to thromboembolic events (El-Chouli et al., 2023). Beyond cardiovascular comorbidities, CHD is associated with neurodevelopmental and neurocognitive dysfunction. This manifests as motor and language deficits during infancy and early childhood, which can persist into adulthood, contributing to cognitive impairment and increased risk of early-onset dementia (Sood et al., 2024). CHD also negatively impacts mental health, and lifetime prevalence of mood and anxiety disorders among individuals with CHD could be as high as 50% (Sood et al., 2024).

Given the significant impact of long-term comorbidities in individuals with CHD, efforts have been made to understand their pathogenesis and clinical outcomes. Recent studies suggest that sleep-disordered breathing (SDB) may contribute to the development of cardiovascular and neurocognitive complications in people with CHD. Obstructive sleep apnea (OSA) is the most common type of SDB and involves repeated airway collapse during sleep (Abbasi et al., 2021). Compared to the general population, individuals with CHD have higher rates of OSA as a result of multiple mechanisms (Cotts et al., 2014; Drake et al., 2020; Harada et al., 2019; Miles et al., 2016; Momcilovic et al., 2024; Sawatari et al., 2015; Stevans et al., 2020). Central sleep apnea (CSA), a less common form of SDB characterized by improper neural control of breathing, also may be more common among individuals with CHD (Momcilovic et al., 2024). It is well-known that SDB contributes to significant morbidity and mortality. Through repeated episodes of intermittent hypoxia and reoxygenation, SDB increases sympathetic activation, oxidative stress, and systemic inflammation, which contributes to the development of cardiovascular disease and structural brain damage (Bradley and Floras, 2009; Lal et al., 2012). In individuals with CHD, SDB may exacerbate pre-existing anatomic vulnerabilities; therefore, their effect on this population warrants careful evaluation. In this review, we examine the prevalence and impact of SDB in individuals with CHD as well as management of SDB in individuals with CHD.

## Methods

**Figure 1:**
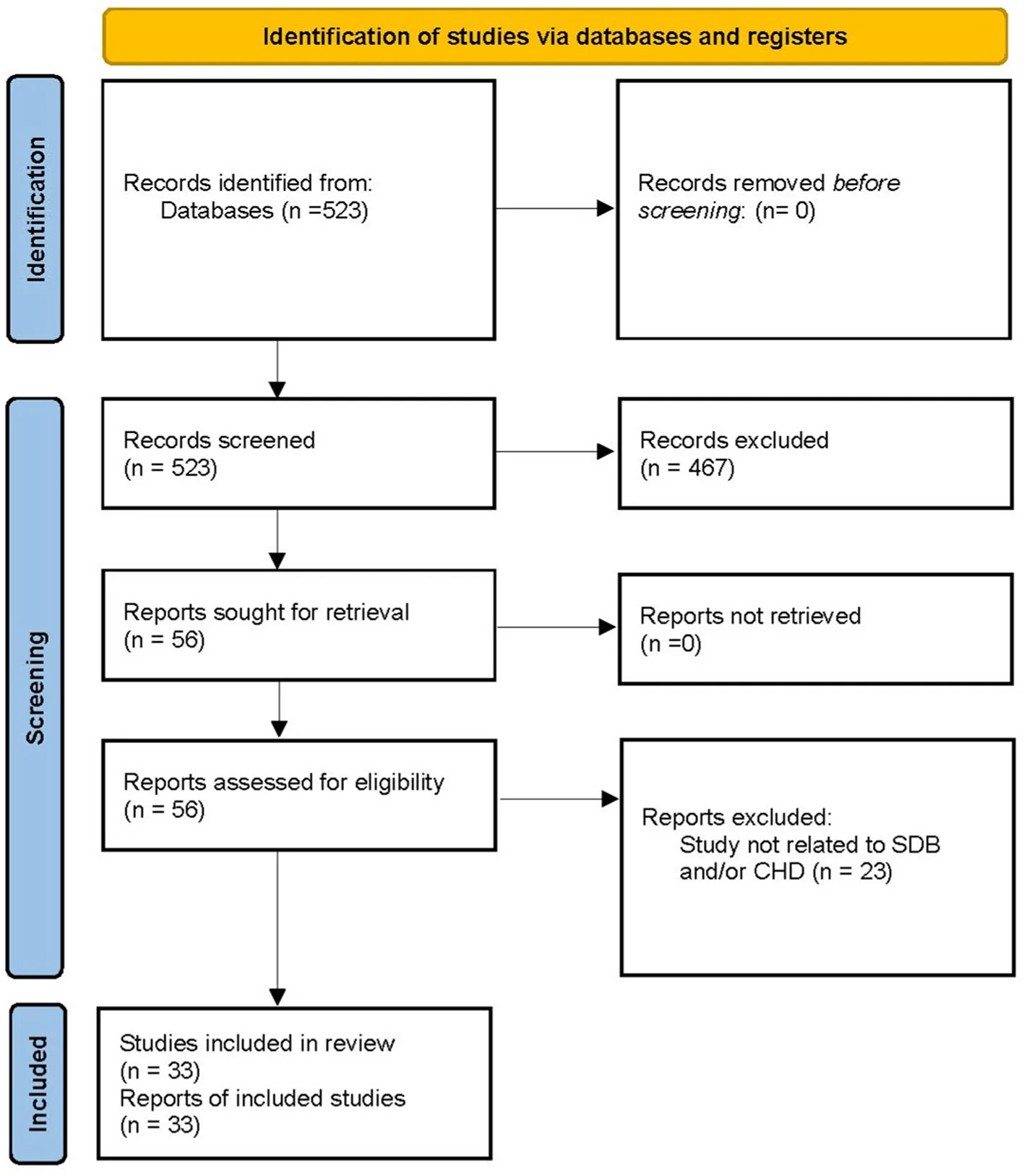
PRISMA flowchart of records screened, sought for retrieval, and assessed for eligibility for inclusion in this review.

We conducted a narrative review of the available literature addressing sleep disorders in the CHD population, with specific attention to SDB. Pubmed, Ovid, Cochrane library database searches from 1990 to 2025 were performed for English-language articles cross-referencing “congenital heart disease” or “Fontan circulation” with pertinent search terms including “sleep,” “sleep disorders,” “sleep disordered breathing,” “sleep quality,” “OSA,” AND either “children” or “adults.” References of included articles were also reviewed to identify additional relevant articles. We identified relevant articles to include in the review based on the inclusion and exclusion criteria. Inclusion criteria: study performed in individuals with CHD; study used objective or self-report tools to assess SDB. Exclusion criteria included articles in another language than English; not an original study containing original data (no protocol articles, conference abstracts, or theses). Two authors screened the titles and abstracts of all identified records to exclude studies that did not meet the eligibility criteria. Any disagreements were resolved by consensus. A total of 523 articles were screened, 56 articles initially identified, and 33 articles met inclusion criteria and are included in this review (Figure 1). Given the narrative nature of this review, the review was not prospectively registered in Prospero. Additionally, given the goal of providing a broad overview of all available studies on SDB in people with CHD and the significant heterogeneity of studies preventing quantitative analysis across studies, no risk of bias assessment was performed.

## Results

### Sleep phenotypes observed in individuals with CHD

Studies in both children and adults with CHD suggest an increased burden of SDB compared to the general population. In cohort studies of adults with CHD, the prevalence of SDB ranges from 26 to 71% (Cotts et al., 2014; Drake et al., 2020; Harada et al., 2019; Miles et al., 2016; Momcilovic et al., 2024; Sawatari et al., 2015; Stevans et al., 2020), significantly higher than the prevalence of 3%−28% observed in the general population (Chowdhuri et al., 2016). Table 1 shows the prevalence of SDB reported across studies. This trend is also present in children. Based on pediatric sleep questionnaire results for a cohort of children with CHD, 38% of screened positive for SDB (Herold et al., 2006). Seventeen of 30 (57%) children age 6–17 years with CHD had OSA on home sleep apnea testing, and a study of infants with CHD found that 11 out of 14 (79%) had SDB on polysomnogram; these values are much higher than the estimated prevalence of 2%−6% in the general pediatric population (Combs et al., 2020; Marcus et al., 2012; Ykeda et al., 2009).

**Table 1:** Results of included studies for prevalence of SDB in individuals with CHD.

Study	Age range	CHD lesion type	Method	SDB prevalence (%)
Harada et al. (2019) (*n* = 104)	Adults	CHD (unspecified)	Overnight polygraph	63
Drake et al. (2020) (*n* = 149)	Adults	CHD (unspecified)	Berlin questionnaire	31
Momcilovic et al. (2024) (*n* = 100)	Adults	CHD (unspecified)	Overnight polygraph	26
Stevans et al. (2020) (*n* = 22)	Adults	Fontan circulation	Polysomnogram	77
Cotts et al. (2014) (*n* = 32)	Adults	d-TGV	STOP questionnaire	44
Miles et al. (2016) (*n* = 22)	Adults	Pulmonary valve incompetence	Overnight oximetry	59
Legault et al. (2008) (*n* = 10)	Adults	Cyanotic CHD	Polysomnogram	0
Herold et al. (2006) (*n* = 37)	Children	Tetralogy of Fallot	Pediatric sleep questionnaire (PSQ)	38
Combs et al. (2020) (*n* = 30)	Children	Corrected moderate to complex CHD	Home sleep testing	57
Ykeda et al. (2009) (*n* = 14)	Infants	CHD (unspecified)	Polysomnogram	79
Sawatari et al. (2015) (*n* = 650)	Children and Adults	CHD (unspecified) and concomitant Down Syndrome	Questionnaire	34.8

The high variance in SDB prevalence is likely due to marked differences in sample demographics (adults vs. children vs. infants as well as varied inclusion of CHD lesions) and study methods (polysomnography vs. home sleep testing vs. questionnaires) that limit direct comparison of studies. All studies except Legault et al. found an increased prevalence of SDB in individuals with CHD compared to general population estimates.

Whereas obstructive sleep apnea (OSA) is the most common form of SDB in the general population, SDB may present more heterogeneously in individuals with CHD. Harada et al. (2019) found that 92% of cases of SDB in individuals with CHD were OSA; however, Momcilovic et al. (2024) reported a more even split between OSA and CSA, with a slight predominance of CSA. Additionally, Stevens et al. observed a significant proportion of individuals with Fontan circulation with nocturnal hypoxemia (Miles et al., 2016; Stevans et al., 2020). Given that baseline hypoxemia is a common feature in Fontan circulation, nocturnal hypoxemia was defined as a desaturation of 5% from baseline oxygen saturation. However, the authors did not specify the duration of these desaturation events, which limits interpretability of the nocturnal hypoxemia results and may result in an elevated estimate of nocturnal hypoxemia in this population. Another study of eight adults with CHD did not identify SDB in any participants, further supporting the existence of significant variation in this population (Legault et al., 2008). Heterogenous SDB presentation is likely also present in children with CHD, which may result in inaccurate results on common screening tests. In a group of 15 infants with CHD, Stamm et al. (2020) found that all had SDB on polysomnography; however, 5 of them would have passed a car seat challenge, a common screening test for cardiorespiratory instability in preterm infants. Overall, these findings indicate that although SDB is common in people with CHD, it is not monolithic, and the substantial heterogeneity in phenotype likely reflects underlying variations in CHD anatomy, individual physiology, and disease severity.

### Cardiovascular physiology as a modifier of sleep disordered breathing

Studies restricted by lesion type give some insight into the pathogenesis of SDB in individuals with specific forms of CHD. In individuals with Fontan circulation, Stevans et al. (2020) found a high prevalence of SDB and significant correlation between low baseline O2 saturation and apnea-hypopnea index (AHI; measure of SDB severity), further enhancing adverse outcomes as it pertains to SDB. Interestingly, BMI and age, which are major risk factors for SDB in the general population, were not significantly correlated with SDB severity in this cohort, suggesting that there are other important factors related to SDB pathogenesis in individuals with Fontan circulation. For instance, chronically decreased cardiac output and elevated central venous pressure can result in lower-extremity edema that redistributes to the neck when laying supine and increase risk for airway obstruction during sleep. Furthermore, individuals with Fontan circulation rely on passive blood flow from the vena cava to the lungs, so they are especially sensitive to increased pulmonary vascular resistance (PVR), a common effect of SDB-induced hypoxia (Stevans et al., 2020). This may explain why individuals with lower baseline O2 saturation, and thus higher PVR have more severe SDB.

Higher rates of SDB have also been observed in individuals with d-transposition of the great arteries (d-TGA) post-atrial switch procedure and those with chronic pulmonary regurgitation (Cotts et al., 2014; Miles et al., 2016). Like with Fontan circulation, the interaction between these lesions and SDB may be reciprocal: cardiovascular dysfunction increases risk of SDB, which in turn worsens the lesion complications. Since the “weaker” morphologic right ventricle serves as the systemic ventricle in d-TGA after atrial switch, it is especially susceptible to systolic and diastolic dysfunction, and the resulting pulmonary edema can increase risk for SDB. Furthermore, the morphologic left ventricle, which supports pulmonic circulation in d-TGA, is susceptible to atrial-ventricular desynchrony (Cotts et al., 2014). This can further contribute to pulmonary vascular congestion, which may compound the increased PVR caused by SDB. A similar phenomenon may occur in individuals with pulmonic regurgitation, as right-sided volume overload can compound the vasoconstrictive effects of SDB on the pulmonary circulation (Miles et al., 2016).

Many studies have observed that individuals with OSA have a higher rate of PFO compared to those without OSA, suggesting a mechanistic link between right-to-left shunting and the development of SDB (Guchlerner et al., 2012; Lau et al., 2010, 2013; Mojadidi et al., 2015; Shaikh et al., 2013; Shanoudy et al., 1998). Notably, individuals with PFO are more likely to display symptoms of OSA at a younger age and have more frequent oxygen desaturations during respiratory disturbances, possibly due to shunting of deoxygenated blood from the right to left atrium during sleep (Johansson et al., 2007; Mojadidi et al., 2015). Additionally, Johansson et al. observed that a larger PFO is associated with a higher degree of nocturnal desaturation, further supporting the hypothesis that right-to-left shunting exacerbates the hypoxemic consequences of OSA (Johansson et al., 2007). Overall, CHD may increase the likelihood of SDB through a variety of lesion-dependent mechanisms, including fluid overload and right-to-left shunting.

### Clinical consequences of sleep disordered breathing in CHD

SDB impacts clinical outcomes across the lifespan of people with CHD. In a retrospective cross-sectional study of inpatient infants with CHD, the presence of SDB was associated with higher total charges, longer inpatient stay, and increased mortality risk (Combs et al., 2018). After infancy, SDB may continue to affect development and function in children with CHD. A retrospective cross-sectional study of children with Fontan circulation found that unspecified sleep disturbances were associated with lower health-related quality of life and increased risk of attention problems, anxiety, and depression (Knobbe et al., 2021). Another study found that children with CHD and OSA scored significantly lower on memory and IQ tests compared to their counterparts without OSA (Combs et al., 2020). In addition to neurodevelopmental problems, SDB may also impact cardiovascular function in children with CHD. Herold et al. (2006) observed that children with CHD who have a positive screen for SDB experience a higher cardiovascular symptom burden, especially palpitations and exercise intolerance. Caissie et al. (2025) found a significant association between sleep quality and adaptive functioning children with CHD but not healthy controls, suggesting that sleep may be especially critical for this population.

**Figure 2:**
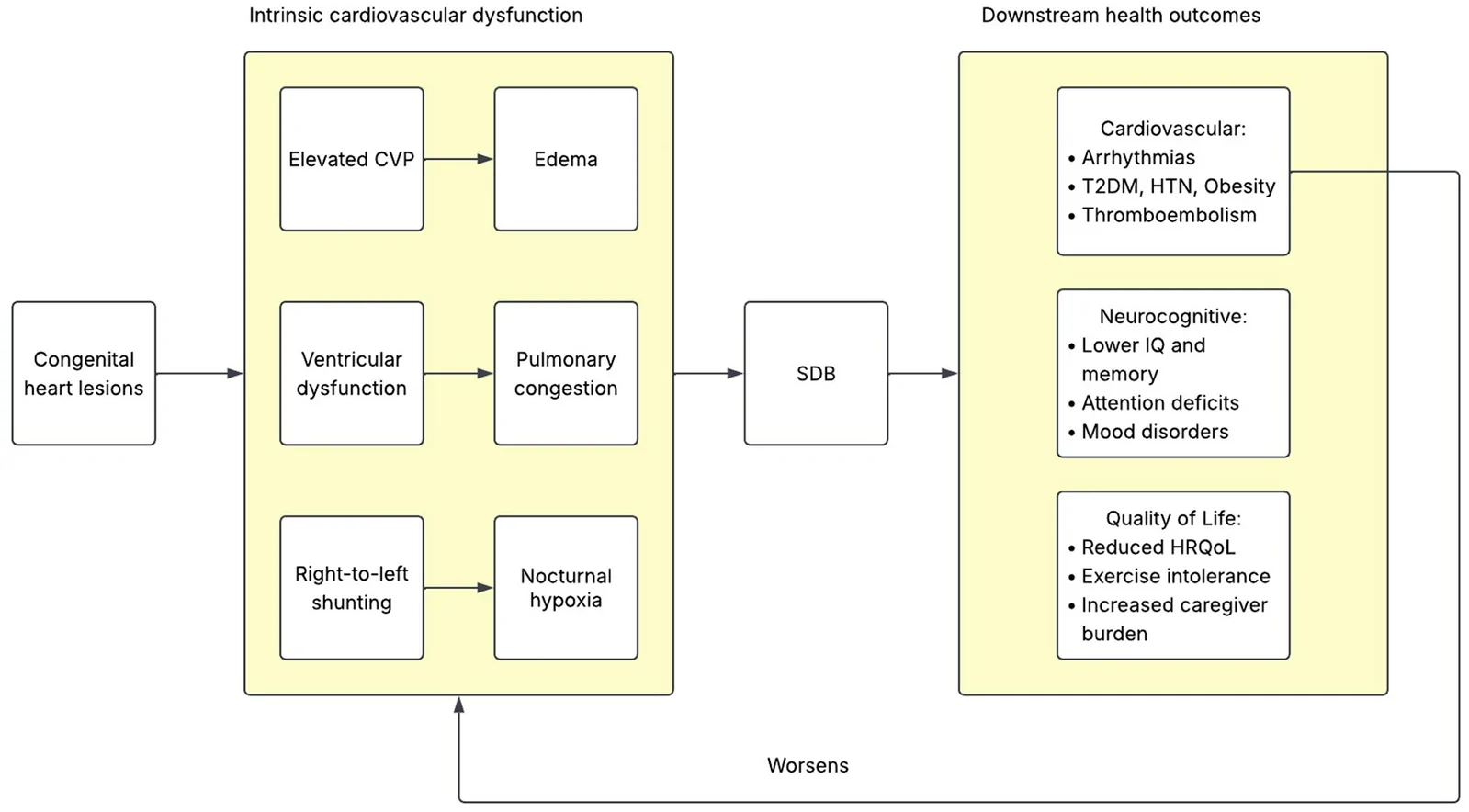
Flowchart showing potential mechanistic links between congenital heart disease (CHD) and sleep disordered breathing (SDB). CHD leads to various forms of cardiovascular dysfunction, such as dependent edema, pulmonary congestion, and nocturnal hypoxia, which may promote the development of SDB or exacerbate its effects. In turn, the downstream effects of SDB may compound the underlying cardiovascular dysfunction from CHD. However, it is important to note that most of the existing literature on SDB in the setting of CHD is observational or cross-sectional, limiting the ability to establish causal relationships between SDB and adverse outcomes in individuals with CHD.

For individuals with CHD, the clinical consequences of SDB persist into adulthood, manifesting as increased risk for cardiovascular and cerebrovascular disease. SDB can compound the effects of pre-existing lesions; for instance, sleep apnea may promote right-to-left shunting through PFOs, increasing risk of paradoxical emboli and stroke (Beelke et al., 2002; Kar, 2014; Pellaton et al., 2011). Additionally, studies have found that OSA is an independent risk factor for arrhythmias such as atrial fibrillation and atrial flutter in adults with CHD (Escudero-Martinez et al., 2023; Jame and Barnes, 2020; Loomba et al., 2017; Vyas et al., 2024). This may further compromise the underlying cardiac dysfunction, worsening the stasis of blood within the heart and promoting thromboembolism formation. SDB in adults with CHD is also associated with diabetes, hypertension, and obesity, which all impair cardiovascular and cerebrovascular function (Drake et al., 2020). Overall, available evidence suggests that the clinical consequences of SDB overlap with and may compound the effects of congenital cardiac lesions across childhood and adulthood, which can be seen in Figure 2.

### SDB therapeutic considerations in select populations with CHD

Due to altered cardiac anatomy and resulting physiologic changes, treatment of SDB in individuals with some forms of CHD may be significantly more complex than SDB treatment for the general population. For instance, individuals with Fontan circulation lack a sub pulmonary ventricle and are especially sensitive to increases in intrathoracic pressure caused by continuous positive airway pressure (CPAP), the gold standard therapy for OSA. Specifically, CPAP leads to increased intrathoracic pressure resulting in increased pulmonary resistance, leading to decreased systemic ventricular preload. As cardiac output in Fontan circulation is exquisitely preload dependent, the increased intrathoracic pressure and increased pulmonary vascular resistance caused by CPAP can reduce systemic cardiac output. Case reports have reported success in using cardiac catheterization, echocardiography, and peripheral venous pressure monitoring to identify a maximum tolerated CPAP pressure, which can guide treatment for individuals with Fontan circulation (Boutsikou et al., 2019; Nanayakkara et al., 2020; Watson et al., 2009). Thus, individuals with CHD can benefit from standard therapies for SDB, such as CPAP, but it is important to individualize treatment parameters according to their physiology.

In addition to ventilation strategies, several studies have also investigated the effects of lesion repair in improving SDB for people with CHD, particularly those with PFO. Since PFO and other forms of right-to-left shunting may exacerbate SDB, investigators have proposed shunt closure as a potential therapeutic strategy. Rimoldi et al. (2015) observed a significant decrease in OSA severity as measured by apnea-hypopnea index (AHI) after transcatheter PFO closure. However, Hoole et al. (2017) and Shaikh et al. (2013) did not find a significant reduction in AHI after PFO closure compared to baseline, though the former did find a decrease in subjective sleepiness. Lesion repair may improve SDB in selected individuals, but the results are inconsistent, suggesting that non-anatomic factors such as ventilatory control and upper-airway physiology may also contribute to the development of SDB in individuals with CHD.

## Discussion

Sleep disorders are a prevalent but heterogenous comorbidity in children and adults with CHD. Both children and adults have demonstrated higher rates of SDB compared to the general population. Notably, individuals with CHD exhibit wider variation in SDB presentation. While OSA accounts for the vast majority of SDB in the general population, a significant proportion of individuals with CHD have CSA or nocturnal hypoxemia without apnea. Some associations between SDB and CHD are straightforward, such as nocturnal hypoxemia in individuals with mixing of systemic and pulmonary circulation leading to a low baseline oxygen saturation. However, the literature examining specific CHD lesions and SDB is mixed with studies showing limited or conflicting associations.

Larger studies are needed to evaluate the underlying mechanisms that appear to increase risk for SDB in people with CHD. One potential mechanism of the increased risk for SDB is that underlying defects in neural crest cell migration underlie both craniofacial developmental abnormalities and CHD (Keyte and Hutson, 2012). Neural crest cells are critical to brain, craniofacial and heart development. Therefore, it is possible that individuals with the phenotype of CHD may also have underlying craniofacial abnormalities predisposing them to SDB. Consistent with this, prior literature has shown that airway anomalies are common on autopsy in children with CHD (Gucer et al., 2005) and an analysis of an administrative dataset found that airway anomalies were reported 20 times more frequently in admitted infants with single ventricle CHD compared to admitted infants without CHD (Kleinsasser et al., 2026).

The wide-ranging physical and mental consequences of sleep disorders are well documented and may be particularly detrimental for people with CHD who are already at risk for health problems, poor quality of life and neurodevelopmental problems. SDB is associated with higher mortality rates in infants with CHD and continues to affect individuals throughout their lifespan and have major impacts on quality of life. Several studies in children and adolescents with CHD have demonstrated an association between poor sleep quality and decreased IQ, memory, attention and adaptive functioning, as well as increased mood problems. Additionally, CHD and sleep disorders can both have profound adverse effects on caregivers and families. There is a paucity of research on caregiver stress and burden for caregivers of individuals with CHD and SDB.

SDB treatments represent a novel approach that may improve the cognitive, behavioral, and quality of life impairments well known to occur in individuals with CHD. Standard treatments for SDB like CPAP or other non-invasive ventilatory modalities can be successful but may require modification for some forms of CHD. For example, because of intrinsic preload dependence, individuals with Fontan physiology are particularly sensitive to increases in PVR and may therefore require lower pressures during CPAP therapy.

Given the complexity of treating SDB in individuals with CHD, novel approaches may be particularly important for individuals with CHD. Recent studies have identified medication treatments including tirzepatide and the combination of atomoxetine and oxybutynin as novel OSA treatments that may be particularly useful for a unique population that has unique physiologic risks limiting CPAP treatment (Combs et al., 2023; Malhotra et al., 2024). However, there are limited existing data on any of these therapies specific to individuals with CHD. The combination of atomoxetine and oxbutynin was studied in children with Down syndrome and 73% of these children had comorbid CHD. However other studies of novel OSA treatments have not specifically included individuals with CHD. Interventional studies including individuals with CHD would be valuable in showing whether these medications can serve as effective alternatives to CPAP for people with CHD.

Further research is needed on optimal treatment strategies for central sleep apnea or nocturnal hypoxemia. Non-invasive ventilation (with the caveats previously noted) or oxygen may be reasonable approaches for central sleep apnea. Oxygen may be safe and effective for central sleep apnea and nocturnal hypoxemia for many individuals with CHD. However, oxygen may also lead to pulmonary overload in certain forms of CHD, particularly unrepaired shunt lesions or palliated cyanotic defects. Similarly, acetazolamide could be considered for central sleep apnea treatment but there is no published evidence for this specific to SDB treatment individuals with CHD. Given often critical balance of volume status for systemic cardiac output and pulmonary overload in individuals with CHD and the diuretic effect of acetazolamide, this may also be contra-indicated for some individuals. Finally, for many forms of CHD, particularly prior to final repair, oxygen saturations below 88 or 90% are expected secondary to the CHD lesion and right to left shunting of de-oxygenated blood. Careful coordination with the individual's cardiologist is needed to avoid unintended consequences of SDB treatment.

The existing literature highlights the importance of SDB in individuals with CHD, however there are significant limitations to the existing literature and further work is needed. Current literature is generally limited to only cross-sectional or observational studies. Many studies included only a small number of participants. Prospective longitudinal studies, ideally across multiple centers, are needed to further understand the impact of SDB on health, quality of life and neurodevelopment in individuals with CHD and other sleep disorders on individuals with CHD. Such studies would also be an ideal opportunity to evaluate other sleep disorders as well, such as insomnia. Existing studies of SDB have used heterogenous approaches to SDB diagnosis including questionnaires, home sleep tests and in-lab polysomnography. This is likely to explain some of the varied estimates of prevalence across studies and the utility of direct comparisons across studies in this review is limited due to this. Consistent diagnostic testing, ideally with in-lab polysomnography would allow for a more accurate assessment of the differences in SDB prevalence across CHD lesions and age groups. Very few studies have more broadly evaluated sleep in individuals with CHD, and these studies have not been designed from the ground up to evaluate sleep outcomes. Most existing data for sleep disorders beyond SDB in people with CHD have been done through secondary analysis of large studies, often with only a limited evaluation of sleep (Knobbe et al., 2021). There is no detailed rigorous broad assessment of sleep and associated impairments in individuals with CHD. An ideal study may include objective sleep testing with polysomnography along with actigraphy or other measures of sleep timing, sleep duration, and perceived sleep quality. Alternatively, a more rigorous assessment of sleep in CHD studies not focused on sleep disorders would likely yield rich data at a very low cost. Incorporating validated sleep questionnaires and a brief survey of sleep timing and duration would require only a few extra minutes from participants.

Overall, current evidence highlights that SDB appears to occur much more frequently and manifests with greater heterogeneity in both children and adults with CHD. SDB has a strong negative association with neurocognition, cardiovascular function, and quality of life for people with CHD. SDB screening for individuals with CHD should be implemented into clinical care pathways. Larger, more rigorous studies of sleep, including SDB–focused clinical trials, are needed in children and adults with CHD. Recognition and treatment of SDB is a promising approach to improve the lives of individuals with CHD and improve outcomes in both the short term and long term.
